# Social prescribing services for patients with musculoskeletal conditions compared with other long-term health conditions in the UK: a cross-sectional study

**DOI:** 10.1136/bmjph-2024-002381

**Published:** 2025-10-05

**Authors:** Qian Gao, Daisy Fancourt, Andrew Steptoe, Feifei Bu

**Affiliations:** 1 School of Public Health, Imperial College London, London, UK; 2 Department of Behavioural Science and Health, Institute of Epidemiology and Health Care, University College London, London, UK; 3 Global Business School for Health, University College London, London, UK

**Keywords:** Public Health, Community Health, Comorbidity

## Abstract

**Introduction:**

To examine the profile of social prescribing (SP) services for patients with long-term conditions (LTCs) and the extent to which equality and benefits of SP services are available to patients with musculoskeletal (MSK) conditions and those with other LTCs.

**Methods:**

This study used routine data from Access Elemental (2017–2022), the most widely used SP software platform in the UK. Our analytical sample was restricted to 4943 patients with LTCs. We used heat maps to map process information about SP services and conducted logistic regression analyses.

**Results:**

The top reason for SP referrals for patients with MSK conditions was pain management, while for other LTCs it was to promote nutrition and diet, support emotional well-being and increase social connectedness and support. However, the top interventions for managing MSK conditions were similar to those prescribed for patients with all other LTCs, targeting activities around promoting diet and nutrition, mental health, physical activity, specific illness support, social connectedness and support. We found that compared with people with no MSK conditions and being referred via non-medical routes, those living with MSK conditions and patients who received referrals from medical centres had lower odds of receiving SP services and having their needs met. Patients living in less deprived areas are more likely to receive SP and have their needs satisfied than those from more deprived neighbourhoods.

**Conclusions:**

SP services do appear to be reaching some patients with MSK conditions, but they are less likely than other patients with LTCs to receive referrals or have their needs met. This suggests that specialist guidance around supporting the needs of patients with MSK conditions through SP is warranted. Further research is also needed to identify whether the broad types of interventions being prescribed to patients with MSK conditions actually meet their initial, most common referral need of pain management.

**WHAT IS ALREADY KNOWN ON THIS TOPIC**

- Social prescribing (SP) is a practical approach to holistically supporting care as a potential solution to tackle inequality in social determinants of health. But there is limited evidence of the equity and benefits of SP for supporting long-term condition (LTC) management.

**WHAT THIS STUDY ADDS**

- The study provided the first estimates on the profiles of SP services (referral resources, reasons for referrals and intervention prescription) for patients with musculoskeletal (MSK) conditions and other LTCs in the UK, using routine data from Access Elemental (2017–2022). We found that the leading reason for patients with MSK conditions is pain management, indicating a different priority to other patients with LTCs, who are most commonly referred for intervention reasons relating to promoting healthy diets, improving emotional well-being and increasing social connectedness and support. Our findings indicate that patients with MSK conditions are less likely than other patients with LTC to receive referrals for SP services or have their needs met.

**HOW THIS STUDY MIGHT AFFECT RESEARCH, PRACTICE OR POLICY**

- Further research is needed to identify how patients with MSK conditions could be more effectively reached through SP and how their needs could be better met through more targeted intervention referrals.

## Introduction

 Musculoskeletal (MSK) conditions refer to a broad range of health conditions affecting joints, muscles, bones and pain syndromes and constitute a major cause of disability.^1^ They are estimated to affect over 494 million people worldwide in 2020 and are projected to double by 2050.^2^ In England, around 20% of general practitioner (GP) consultations annually are MSK related, and MSK conditions are the leading causes of sickness absence.^3^ Arthritis and other MSK conditions are common with advancing age, and among females and those with lower socioeconomic status,^4 5^ MSK conditions are frequent contributors to multiple long-term conditions (LTCs). People with LTCs have greater and more complex health needs.^6^

In recent years, there has been increasing recognition that non-clinical interventions can help to supplement continuity of care for people with MSK conditions and other LTCs.^7^ Social prescribing (SP) is a mechanism of care that has been increasingly used over the past decade as an important adjunct to conventional care to connect patients to such interventions to improve health and well-being outcomes.^7 8^ It is a key component in delivering universal personalised care and part of the National Health Service (NHS) England long-term plan,^9^ as well as healthcare policies extended to other devolved nations of the UK.^10^,^12^ The predominant SP model in the UK comprises a healthcare professional referring patients to a link worker (or community connector, community navigator, health trainer, etc) who then works with the patient to develop a personalised care plan which connects them to community support and non-medical interventions,^7^ with activities falling into four main categories: (1) providing advice and information and supporting engagement with (2) the arts and heritage, (3) the natural environment and (4) physical activity.^13^

Evidence from local evaluations and preliminary trials points towards SP helping with a wide range of problems, including supporting mental health, well-being and health behaviours^14 15^ and managing MSK conditions.^16^ Preliminary evidence also suggests that SP may reduce healthcare demand and costs or move healthcare demand to primary care services rather than more expensive tertiary care, demonstrating a favourable return on investment.^17^ Given its potential, NHS England has committed to tripling the number of link workers by 2036/2037.^18^ However, despite the promises of SP, national evaluations remain scant. Reviews into the use of SP suggest that referrals are not equitable, with those who are aged under 16 years, male and those from non-white backgrounds being less likely to be offered SP.^19^ There are even concerns that SP may not be effective for addressing social inequalities in health.^20 21^

To date, there is limited research into whether SP supports the health management of people with MSK conditions.^22^ To bridge this evidence gap, this study provided insight into the utilisation of SP services for patients with MSK conditions by exploring the profiles of SP services, including (1) referral resources, (2) referral reasons and (3) prescription details of SP interventions (eg, numbers and types of intervention prescriptions). We specifically compared the experiences of MSK conditions with patients with other LTCs. We also examined the factors associated with receipt of SP and patient need satisfaction after using SP services among patients with MSK conditions and other LTCs (ie, chronic obstructive pulmonary disease (COPD), common mental disorders (CMDs), diabetes, physical disabilities, asthma and others).

## Materials and methods

### Data sources

The study used routine data obtained from Access Elemental,^23^ a digital SP platform used by health and social care professionals, community development workers and other service providers to keep track of SP activities and their impact starting from the point of referral. Elemental is the largest digital SP platform in the UK, with excellent geographical coverage. To date, data are collected from over 800 social prescribing hubs spread across the UK, representing 16 000 prescribers (including GPs, nurses, practice managers, housing officers, local government staff and social workers) from 48 primary care networks and 287 GP practices. This large SP database contains information about patients’ sociodemographic characteristics, health conditions, prescriptions, interventions and use of SP services. There were 201 037 SP cases from 169 818 unique individuals in the Elemental dataset (January 2017–November 2022). 94.3% of individuals provided valid postcodes (n=160 128), allowing for data linkage with publicly available geographic information. There were 4943 patients in the dataset who were reportedly diagnosed with at least one LTC (including MSK), constituting our analytical sample.

### Measures

We quantified a retrospective cohort of patients with MSK conditions and other LTCs in the Elemental dataset. Patients with MSK conditions were those who had osteoarthritis, arthritis, osteoporosis, musculoskeletal and broader chronic pain conditions,^2^ and those who had referral reasons to SP services due to MSK symptoms (eg, osteoarthritis, arthritis, osteoporosis, musculoskeletal pain and chronic pain). A list of LTCs was measured through self-reported medical history, including COPD, diabetes (prediabetes and type 1 and 2 diabetes), physical disability, asthma, common mental disorders (eg, depression and anxiety) and other LTCs (ie, cancer, dementia, development or intellectual disability, coronary heart disease (including high blood pressure), high cholesterol, learning disability, neurological conditions, sensory impairment, stroke/transient ischaemic attack and others). The study separately reported the individual category of LTCs for those relatively comparable to MSK conditions with enough cases (at least 5% of the sample reporting it). The rest of the LTCs were reported in the ‘other LTCs’ category.

The study considered outcome measures of SP services, including (1) whether or not receiving prescriptions of SP services (yes vs no) and (2) patients’ perceived needs satisfaction after engaging in SP (yes vs no (unmet/no longer requires service/unclear)) through self-report. We included the process information about SP services in relation to reasons for referrals (eg, pain, mental health, social isolation, etc) and referral resources (medical (primary/secondary care) versus non-medical (eg, education, local authority and self-referral)), along with intervention prescription (eg, talking therapies, welfare/financial support, volunteering, exercise programmes, arts/cultural activities, etc). Our analysis also included a series of demographic information on age, gender, ethnicity, country (England, Wales, Scotland and Northern Ireland), living areas (urban vs rural) and Index of Multiple Deprivation (IMD; quintiles, 1—most deprived to 5—least deprived) via postcode linkage to governmental statistics in each country. IMD is a statistical proxy of relative neighbourhood deprivation derived for small areas, which combines weighted domains of deprivation (ie, income; employment; education, skills and training; health deprivation and disability; crime; barriers to housing and services and living environment deprivation).^24^

### Statistical analysis

In this cross-sectional study, we first conducted descriptive analyses to assess and present the sociodemographic characteristics and health-related variables of samples with MSK conditions and other LTCs. We then investigated and compared the profiles of SP services (ie, top presenting reasons for referrals to SP, types of interventions, prescription and referral resources) across different health conditions. Binary logistic regression models were conducted to assess the correlates of receiving SP services and patients’ need satisfaction, involving age, gender, IMD, living area, MSK conditions and referral source. Under the assumption of missing at random, we applied multiple imputation by chained equations to address missing data,^25^ and 35 imputed datasets were generated and combined using Rubin’s rule (online supplemental etables 1 and 2).^26^ All statistical analyses were performed using Stata V.18.0.^27^

## Results

### Participants

Among 4943 patients with LTCs, 1183 patients had MSK conditions (mean age=63.6, SD=16.6), and 3760 patients had other LTCs (mean age=59.6, SD=17.7; table 1). Over half of the patients with MSK conditions (61.6%) and 50.4% of patients with other LTCs were 60 years and above. Over three-quarters (76.6%) of patients with MSK conditions and 60.8% of patients with other LTCs were female. Most patients with MSK conditions (84.5%) and patients with other LTCs (87.7%) were of white ethnicity, which was similar to national statistics.^28^ 9.8% of patients with MSK conditions and 2% of those with other LTCs were in the lowest quintile of the IMD, while the rest of the samples were about evenly distributed in higher quintiles. Patients with MSK conditions were mainly from England (90%), followed by Northern Ireland (5.2%), Scotland (4.2%) and Wales (0.6%), and the other LTC group consists exclusively of individuals from England.

**Table 1:** Sociodemographic characteristics, social prescription and outcomes among MSK and other long-term health conditions (n=4943)

Characteristics	MSK (n=1183)	Other long-term conditions (n=3760)
Age	63.6 (SD=16.6)	59.6 (SD=17.7)
Age group		
<30	37 (3.1%)	224 (6%)
30–59	418 (35.3%)	1637 (43.6%)
≥60	728 (61.6%)	1895 (50.4%)
Gender		
Female	900 (76.6%)	2263 (60.8%)
Male	275 (23.4%)	1459 (39.2%)
Ethnicity		
White	317 (84.5%)	1376 (87.8%)
Black/African-Caribbean	21 (5.6%)	47 (3%)
Asian (including Indian, Chinese, Pakistani, etc)	28 (7.5%)	129 (8.2%)
Others (including mixed ethnicity)	9 (2.4%)	15 (1%)
Country		
England	1031 (90%)	3697 (100%)
Wales	7 (0.6%)	0
Scotland	48 (4.2%)	0
Northern Ireland	60 (5.2%)	0
Living areas		
Urban	889 (85.6%)	3144 (85%)
Rural	149 (14.4%)	553 (15%)
Index of Multiple Deprivation (quintiles)		
Q1 most deprived	101 (9.8%)	76 (2%)
Q2	208 (20.2%)	446 (12.1%)
Q3	237 (23%)	865 (23.4%)
Q4	289 (28%)	1282 (34.7%)
Q5 least deprived	196 (19%)	1028 (27.8%)
Numbers of SP prescriptions (mean (SD))	3.4 (2.5)	3.7 (2.6)
Numbers of SP prescriptions (ranges)	0–23	0–22
Referral source		
Medical centres (primary care)	771 (86.5%)	2086 (85%)
Community (including self-referral)	120 (13.5%)	368 (15%)
SP prescriptions		
Yes	683 (57.7%)	2634 (70.1%)
No	500 (42.3%)	1126 (29.9%)
Patients’ needs met		
Needs met	515 (43.5%)	2150 (57.2%)
Unmet/no longer requires service/unclear	668 (56.5%)	1610 (42.8%)

MSK, musculoskeletal; SP, social prescribing.

A slightly higher percentage of patients with MSK conditions were referred from medical routes (eg, primary or secondary care) compared with patients with other LTCs (86.5% vs 85%). Among patients with MSK conditions, 57.7% of the samples received SP intervention prescriptions (mean=3.4, SD=2.5). In contrast, 70.1% of patients with other LTCs had any intervention prescribed (mean=3.7, SD=2.6). 43.5% of patients with MSK conditions reported having their needs met after receiving SP, compared with 57.2% for patients with other LTCs.

### Top reasons for referrals among patients with MSK conditions and other LTCs

The details of the top referral reasons are shown in table 2. Top reasons for referrals among patients with MSK conditions (n=1183) were ‘pain management/chronic pain’ (23.8%), ‘weight management/healthy eating’ (19.4%), ‘support with emotional well-being’ (12.2%), ‘become more active’ (10.1%), ‘improving social contact’ (7.7%), ‘MSK/arthritis’ (4.4%) and ‘maintaining independent living’ (4.2%). The top reasons were shown to be similar among patients with MSK conditions and those with other LTCs, except for pain management. In those with CMDs (n=1581), the top reasons for referral included ‘support with emotional well-being’ (35%) and ‘weight management/healthy eating’ (31.6%), followed by ‘become more active’ (9.1%) and ‘improving social contact’ (6.1%). Likewise, the top reasons for referral were the same among those with COPD (n=215), including ‘weight management/healthy eating’ (23.3%), followed by ‘support with emotional well-being’ (22.3%), ‘improving social contact’ (13.5%) and ‘becoming more active’ (10.7%).

**Table 2:** Top reasons for referrals among MSK and other long-term health conditions

Reasons	MSK (n=1183)	Chronic obstructive pulmonary disease (n=215)	Diabetes (n=555)	Common mental disorders (n=1581)	Physical disability (n=300)	Asthma (n=411)	Other long-term conditions (n=1102)
Pain management/chronic pain	23.8%	0%	0%	0.4%	0.3%	0%	0.7%
Weight management/healthy eating	19.4%	23.3%	62.7%	31.6%	40%	44%	47.4%
Support with emotional well-being	12.2%	22.3%	11.2%	35%	12.7%	20.2%	13.3%
Become more active	10.1%	10.7%	11.5%	9.1%	13.7%	12.9%	11.3%
Improving social contact	7.7%	13.5%	3.4%	6.1%	5%	4.4%	5.2%
MSK/arthritis	4.4%	0%	0%	0%	0%	0%	0%
Maintaining independent living	4.2%	3.3%	1.4%	1.8%	5%	1.5%	3%
Mental health	3.2%	0%	0.	0.4%	0.3%	0%	0.1%
Carer support	3.1%	3.7%	1.4%	2.8%	3.7%	1.7%	4.3%
Long-term condition management	2.5%	0%	0%	0%	0%	0%	0.1%
Falls prevention/staying mobile	2.2%	1.4%	0.9%	0.6%	2%	1.2%	1.5%
Others	2.1%	3.7%	2.7%	2.3%	6%	2.7%	3.4%
Housing	1.5%	0.9%	0.4%	2.2%	0.7%	1.2%	0.1%
Finances and benefits	1.3%	5.1%	1.8%	3.5%	2%	3.2%	3%
Support with community or social care	1.3%	0.9%	0.9%	0.7%	1%	1%	1%
Support with stopping smoking	0.3%	8.8%	1.3%	1.3%	1.3%	3.9%	1.8%
Alcohol/substance misuse support	0.3%	0.9%	0.2%	1.7%	1%	1%	1.3%
Healthy lifestyle (in general)	0.3%	0%	0%	0%	0%	0%	0%
Physical health support	0%	0%	0%	0.3%	5%	0%	1.9%
COVID-19	0%	1.4%	0.2%	0.6%	0.7%	1.2%	1.4%

MSK, musculoskeletal.

### Intervention prescriptions received by patients with MSK conditions and other LTCs

The top intervention prescriptions for patients with MSK conditions were similar to those of patients with all other LTCs (table 3 and online supplemental figure 1 and table 3). The most common intervention prescription for patients with MSK conditions was ‘social support’ (22.5%), followed by ‘diet and nutrition’ (18.1%), ‘physical activity and exercise’ (15.6%), ‘mental health’ (14.8%), ‘help with independent living’ (11%), ‘specific illness support’ (9.6%) and ‘carer support’ (9.6%). Likewise, these intervention prescriptions were among the high-ranked prescriptions for patients with other LTCs.

**Table 3:** Top intervention prescriptions for patients with MSK and other long-term health conditions

Prescriptions	MSK (n=1183)	Chronic obstructive pulmonary disease (n=215)	Diabetes (n=556)	Common mental disorder (n=1584)	Physical disability (n=300)	Asthma (n=412)	Other long-term conditions (n=1102)
Social support	22.5%	29.3%	31.3%	24.9%	29%	26.5%	25.7%
Diet and nutrition	18.1%	19.1%	44.1%	23%	30.3%	32.8%	32.8%
Physical activity and exercise	15.6%	14%	26.3%	19.2%	20.7%	21.8%	18.8%
Mental health	14.8%	19.5%	18.9%	26.6%	16.7%	20.6%	16.3%
Help with independent living	11%	14%	14.6%	11.5%	12.3%	13.3%	13.4%
Specific illness support	9.6%	17.7%	26.8%	14.1%	20.7%	16%	20%
Carer support	9.6%	13%	14.2%	10.5%	11.7%	11.9%	12.2%
Community activities (general)	8.8%	10.7%	15.6%	11%	13.3%	12.1%	12.2%
Financial and benefits support	7.6%	10.2%	14.4%	10.9%	13%	11.7%	12.1%
Housing support	7.5%	7%	13.1%	9.3%	10.3%	11.4%	10.7%
Alcohol and substance misuse support	6.7%	9.3%	14.9%	10.2%	12%	12.9%	12.4%
Learning and development	6.4%	7%	13.8%	9%	12%	10.4%	10.7%
Smoking support	6.3%	14%	15.1%	10.2%	10.3%	14.1%	11.8%
Weight management	1.2%	2.3%	2.2%	1.8%	1.7%	2.9%	1.9%

LTCs, long-term conditions; MSK, musculoskeletal.

### Factors associated with receipt of SP prescriptions and patients’ perceived need satisfaction after receiving SP services

Patients with MSK conditions had lower odds of receiving SP prescriptions (OR _SP_=0.61, 0.53–0.70) and need satisfaction (OR _NS_=0.60, 0.52–0.69) than patients with other LTCs. Referrals via medical routes were associated with lower odds of receiving prescriptions of SP services (OR _SP_=0.38, 0.30–0.48) and needs being met (OR _NS_=0.42, 0.34–0.51) than being referred via non-medical routes. Compared with younger people under 30, middle-aged (30–59 years, OR _SP_=1.54, 1.18–2.02, OR _NS_=1.64, 1.25–2.15) and older patients (60–100 years, OR _SP_=1.67, 1.28–2.19, OR _NS_=2.04, 1.56–2.66) had higher odds of receiving SP and need satisfaction (table 4). Living in less deprived areas was associated with higher odds of receiving SP prescriptions and need satisfaction. In contrast, patients living in urban areas had lower odds of receiving SP prescriptions (OR _SP_=0.80, 0.66–0.98) and need satisfaction (OR _NS_=0.71, 0.59–0.85).

**Table 4:** Factors associated with receipt of SP and NS among patients with LTCs (n=4943)

Factors	SP prescriptions	NS
LTCs		
Other LTCs	1	1
MSK	0.61 (0.53–0.70)	0.60 (0.52–0.69)
Referral resource		
Non-medical routes	1	1
Medical routes	0.38 (0.30–0.48)	0.42 (0.34–0.51)
Age		
<30 years	1	1
30–59	1.54 (1.18–2.02)	1.64 (1.25–2.15)
60–100	1.67 (1.28–2.19)	2.04 (1.56–2.66)
Gender		
Male	1	1
Female	1.09 (0.95–1.24)	1.03 (0.91–1.16)
Living areas		
Rural	1	1
Urban	0.80 (0.66–0.98)	0.71 (0.59–0.85)
IMD		
Q1	1	1
Q2	1.32 (0.92–1.88)	1.80 (1.24–2.62)
Q3	1.68 (1.20–2.35)	2.03 (1.42–2.90)
Q4	1.91 (1.37–2.66)	2.49 (1.75–3.54)
Q5	1.80 (1.29–2.52)	2.48 (1.74–3.55)

Models adjusted for age, gender, living areas, IMD, MSK condition and referral source.

IMD, Index of Multiple Deprivation; LTCs, long-term conditions; MSK, musculoskeletal; NS, need satisfaction; Q, quintile.

## Discussion

This study is the first to estimate the equality of SP and needs satisfaction among patients with MSK conditions compared with other LTCs. We found that patients with MSK conditions had a different leading reason for referrals compared with patients with other LTCs, but the interventions both groups received were very similar, indicating little targeting of SP to MSK conditions. Furthermore, compared with other LTC groups, patients with MSK conditions had lower odds of receiving SP interventions and having their needs satisfied after receiving SP.

For patients with MSK conditions, the lead referral reason is related to chronic pain management. This is unsurprising given not only that pain is the most prominent symptom for most patients with MSK conditions but also that referral reasons (including pain) were used as one of the ways to identify patients with MSK conditions. But like patients with other LTCs, patients with MSK conditions are also commonly referred to SP due to reasons related to lifestyle (eg, weight management/healthy eating and becoming more active) or emotional and social well-being, some of which could be closely linked to MSK conditon. For instance, poor nutrition, obesity and physical inactivity are among the major risk factors for MSK conditions.^29^ Patients with MSK conditions are also prone to low levels of physical activity due to avoidance or suppressive behaviours.^30^ Furthermore, it has been shown that having an MSK condition is associated with poor mental health and a higher level of loneliness.^31^,^33^ Therefore, it is important to recognise that patients with MSK conditions might have diverse needs in addition to pain, many of which fall within the remit of SP.

In general, the interventions prescribed to patients with MSK conditions are similar to those with other LTCs, with the most common interventions being social support, diet and nutrition, physical activity and exercise and mental health. We see that many of these are in line with referral reasons. It is notable, however, that less than 10% of patients were prescribed specific illness support, which is less compared with other conditions, for example, 26.8% for diabetes and 20.7% for physical disability. This could signify a lack of community resources available to support specific MSK challenges. Although physical activity is recognised as one of the best non-clinical interventions for many MSK conditions to reduce pain,^34^ the percentage of patients referred to physical activity and exercise is largely similar to other conditions. It is likely that it serves as a generic intervention rather than targeting the pain. Therefore, there could be a mismatch between pain as the lead referral reason and the lack of targeted interventions (eg, pain management is not on the menu for SP) for patients with MSK conditions. Besides, many patients with MSK conditions have been offered physiotherapy in the past, and referrals to SP interventions that involve common components, such as physical activity programmes, are not able to fully address their needs. This may explain, to some extent, why patients with MSK conditions are less likely to report having their needs satisfied. Further qualitative research may need to explore the way of fitting SP into patients with MSK conditions’ treatment pathways.

More broadly, we have also found other predictors of not receiving interventions among people with LTCs that were even stronger than MSK diagnoses, such as being referred from medical routes. Patients whose symptoms are difficult to manage may have more unmet health needs,^35^ potentially leading to fewer prescriptions of non-clinical interventions. Although patients who have referrals from medical routes may have more severe health conditions and/or lower acceptability of SP, there is also a need to enhance the coordination between healthcare and SP services. Besides, our findings suggest that those living in less deprived areas are more likely to receive SP interventions than those from the most deprived areas. This may be due to inequalities in the availability of community activities for patients to be referred to, due to the deprivation levels of areas. It is important to improve the uptake and maintenance of non-clinical sociopsychological interventions,^36^ especially for those living in socioeconomically deprived areas, as many face more barriers to SP and are at higher risks of poor health outcomes.

SP is a potentially effective mechanism to provide a non‐clinical person-centred approach to care for individuals with complex health needs.^7^ Our findings indicate that middle-aged and older patients are more likely to have their needs satisfied after receiving SP, implying the necessity of further shaping SP services with particular consideration of differences in patients’ needs across life stages. Although previous research suggested that individuals in high-deprivation areas have the potential to benefit more in mental well-being from social engagement,^37^ we found that patients living in areas of higher deprivation have a lower likelihood of need satisfaction after receiving SP services. Consistent with the key debates around the effectiveness of SP in addressing social inequalities,^20 21^ this may be driven by the availability and quality of SP services across area deprivation, but more in-depth investigations are needed to understand the underlying reasons. Furthermore, patients who received referrals from medical routes (eg, primary care) tended to have a lower likelihood of their needs being satisfied than those referred via non-medical routes. Differences in the severity of diseases could, to some extent, affect positive patient activation, which is closely associated with self-management behaviours^38^ and subsequently shapes patients’ perceived benefits and needs for SP services.

To our knowledge, our study provides the first estimates of SP for patients with MSK conditions and other LTCs and initial evidence about factors associated with receiving SP interventions and their effectiveness (ie, patients’ need satisfaction). Although the findings of this study provide valuable estimates of SP to patients living with LTCs, there were some limitations. First, we identified patients using the records of those who have a self-reported medical history of specific LTCs and further enlarged the samples of patients with MSK conditions through records of referral reasons in relation to MSK (eg, osteoarthritis, arthritis, osteoporosis, musculoskeletal pain and chronic pain). However, the measure of MSK condition and other LTCs was not validated by health records, as linked data were not available to us. We also focused on only a small subsample of the Elemental dataset that had reported full information on LTCs. It is highly likely that other people in the broader dataset also had LTCs that were not reported. We recommend mandatory reporting of LTCs for professionals using the Elemental database to provide augmented sample sizes for future similar analyses. Our sample was restricted to those who had an LTC recorded in the Elemental dataset, resulting in a relative sample imbalance across the UK compared with the national population distribution. This likely reflects differences in data collection practices across the UK and may affect the generalisability of the findings. We acknowledge that the process indicators of SP services (eg, reasons for referrals and intervention prescriptions) were recorded according to individual LTCs in the database and thus limit the understanding of SP services for multiple LTCs. However, there is no reason to assume that this problem would differentially affect people with MSK conditions compared with other LTCs. So our comparative findings in MSK conditions versus other LTCs still hold value. The amount of missingness on referral sources could further challenge the comparisons between medical and others. For these reasons, though, we did not conduct comparisons between MSK conditions and people not reporting any LTCs. Our cross-sectional designs limit the possibility of understanding the longitudinal effectiveness of SP services for addressing the health needs of patients with LTCs, as well as the active ingredients of SP interventions. It is necessary to estimate social prescription adherence and link the SP database with electronic health records to further assess the long-term health benefits of SP and its potential impacts on the health system. Future qualitative research is also needed to explore barriers and facilitators to accessing SP services to inform reforms on improving the accessibility and equity of SP, aiming to better address the health needs of patients living with MSK conditions and other LTCs.

## Conclusions

Overall, our analysis shows that the leading reason for patients with MSK conditions is pain management, indicating a different priority to other patients with LTCs, who are most commonly referred for intervention reasons relating to promoting healthy diets, improving emotional well-being and increasing social connectedness and support. However, despite differences in the referral reasons, SP interventions for patients with MSK conditions and those with other LTCs were similar, frequently targeting activities around promoting diet and nutrition, mental health, physical activity, specific illness support, social activation and support. It is notable that patients with MSK conditions were less likely than other patients with LTC to report their needs being satisfied by SP, suggesting that these broad interventions may not be fully addressing their pain. Further research is needed to identify how patients with MSK conditions could be more effectively reached through SP and how their needs could be better met through more targeted intervention referrals. More broadly, our study highlights the necessity of improving the equality of SP services to ensure that those from areas of higher deprivation and coming from medical referral routes can have equal experiences with other patients. Such work is important to improve the equity and efficiency of SP and ensure it provides the best support it can for individuals with complex health needs.

## Supplementary material

10.1136/bmjph-2024-002381 online supplemental file 1

## Data Availability

Data may be obtained from a third party and are not publicly available.
