# Supplementary material for: Social prescribing services for patients with musculoskeletal conditions compared with other long-term health conditions in the UK: a cross-sectional study
Source: BMJ Public Health. 2025 Oct 5;3(2):e002381. doi: 10.1136/bmjph-2024-002381 (PMC12506216; doi:10.1136/bmjph-2024-002381)
Supplement: online supplemental file 1 [file bmjph-3-2-s001.docx]

**Supplementary materials for: Social prescribing services for patients with musculoskeletal conditions compared with other long-term health conditions in the UK**

**eFigure 1.** Top Intervention prescription for patients with long term conditions (n=4,943)

**eTable 1.** Missingness in sociodemographic characteristics, social prescribing, and outcomes among study samples (n=4,943)

**eTable 2.** Factors associated with patients’ need satisfaction and receipt of social prescribing (complete case n=3,177)

**eTable 3.** Top intervention prescription for patients with MSK and other long term health conditions (n=4,943)

**eFigure 1. Top Intervention prescription for patients with long term conditions (n=4,943)**

**eTable 1. Missingness in sociodemographic characteristics, social prescribing, and outcomes among study samples (n=4,943)**

| **Characteristics** | **MSK**  **(n=1,183)** | **Other LTCs**  **(n=3,760)** |
| --- | --- | --- |
| Age | 0 | 4 (0.1%) |
| gender | 8 (0.7%) | 38 (1.0%) |
| Ethnicity | 808 (68.3%) | 2,193 (58.3%) |
| Country | 37 (3.1%) | 63 (1.7%) |
| Area | 145 (12.3%) | 63 (1.7%) |
| Index of multiple Deprivation (Quintiles) | 152 (12.9%) | 63 (1.7%) |
| Numbers of prescription (mean [SD]) | 0 | 0 |
| Numbers of prescription (ranges) | 0 | 0 |
| Referral source | 292 (24.7%) | 1,306 (34.7%) |
| Prescriptions | 0 | 0 |
| Patients’ needs met | 0 | 0 |

**eTable 2. Factors associated with patients’ need satisfaction and receipt of social prescribing (complete case n=3,177)**

| **Factors** | **Needs satisfaction** | **Social prescribing** |
| --- | --- | --- |
| Age (ref. <30) | 1.0 | 1.0 |
| 30-59 | 1.76 (1.24-2.50) | 1.50 (1.08-2.07) |
| 60-100 | 2.44 (1.17-3.46) | 1.67 (1.21-2.31) |
| Female (ref. male) | 1.03 (0.88-1.20) | 1.17 (1.00-1.36) |
| IMD (ref. Q1) | 1.0 | 1.0 |
| Q2 | 1.44 (0.94-2.20) | 1.13 (0.76-1.68) |
| Q3 | 1.71 (1.14-2.58) | 1.57 (1.08-2.29) |
| Q4 | 2.05 (1.37-3.07) | 1.61 (1.11-2.33) |
| Q5 | 1.85 (1.23-2.79) | 1.40 (0.96-2.04) |
| Urban area (ref. rural) | 0.73 (0.58-0.91) | 0.77 (0.61-0.97) |
| MSK (ref. other LTC) | 0.67 (0.56-0.80) | 0.59 (0.50-0.70) |
| Referral by medical centre  (ref. other/self-referral) | 0.35 (0.28-0.43) | 0.39 (0.31-0.49) |

**eTable 3. Top intervention prescription for patients with MSK and other long term health conditions (n=4,943)**

|  | Types | MSK  (n=1,183) | Types | COPD (n=215) | Types | Diabetes (n=556) | Types | CMH  (n= 1,584) |
| --- | --- | --- | --- | --- | --- | --- | --- | --- |
| 1 | Diet & Nutrition | 18.1% | Mental Health | 19.5% | Diet & Nutrition | 44.1% | Mental Health | 26.6% |
| 2 | Physical Activity & Exercise | 15.6% | Diet & Nutrition | 19.1% | Specific Illness Support | 26.8% | Diet & Nutrition | 23.0% |
| 3 | Mental Health | 14.8% | Specific Illness Support | 17.7% | Physical Activity & Exercise | 26.3% | Physical Activity & Exercise | 19.2% |
| 4 | Social Support | 12.9% | Befriending & Social Isolation | 16.3% | Mental Health | 18.9% | Specific Illness Support | 14.1% |
| 5 | Help with Independent Living | 11.0% | Help with Independent Living | 14.0% | Befriending & Social Isolation | 16.2% | Befriending & Social Isolation | 13.2% |
| 6 | Carer Support | 9.6% | Physical Activity & Exercise | 14.0% | Community Activities - General | 15.6% | Social Support | 11.7% |
| 7 | Befriending & Social Isolation | 9.6% | Smoking Support | 14.0% | Smoking Support | 15.1% | Help with Independent Living | 11.5% |
| 8 | Specific Illness Support | 9.6% | Carer Support | 13.0% | Social Support | 15.1% | Community Activities - General | 11.0% |
| 9 | Community Activities - General | 8.8% | Social Support | 13.0% | Alcohol & Substance Misuse Support | 14.9% | Financial and Benefits Support | 10.9% |
| 10 | Financial and Benefits Support | 7.6% | Community Activities - General | 10.7% | Help with Independent Living | 14.6% | Carer Support | 10.5% |
| 11 | Housing Support | 7.5% | Financial and Benefits Support | 10.2% | Financial and Benefits Support | 14.4% | Smoking Support | 10.2% |
| 12 | Alcohol & Substance Misuse Support | 6.7% | Alcohol & Substance Misuse Support | 9.3% | Carer Support | 14.2% | Alcohol & Substance Misuse Support | 10.2% |
| 13 | Learning and Development | 6.4% | Housing Support | 7.0% | Learning and Development | 13.8% | Housing Support | 9.3% |
| 14 | Smoking Support | 6.3% | Learning and Development | 7.0% | Housing Support | 13.1% | Learning and Development | 9.0% |
| 15 | Health & wellbeing | 2.5% | COVID-19 | 2.3% | Weight Management | 2.2% | Employment | 2.7% |

**eTable 3 (continue) Top intervention prescription for patients with MSK and other long term health conditions (n=4,943)**

|  | Types | Physical disability  (n=300) | Types | Asthma  (n=412) | Types | Others  (n=1102) |
| --- | --- | --- | --- | --- | --- | --- |
| 1 | Diet & Nutrition | 30.3% | Diet & Nutrition | 32.8% | Diet & Nutrition | 32.8% |
| 2 | Physical Activity & Exercise | 20.7% | Physical Activity & Exercise | 21.8% | Specific Illness Support | 20.0% |
| 3 | Specific Illness Support | 20.7% | Mental Health | 20.6% | Physical Activity & Exercise | 18.8% |
| 4 | Mental Health | 16.7% | Specific Illness Support | 16.0% | Mental Health | 16.3% |
| 5 | Befriending & Social Isolation | 15.3% | Befriending & Social Isolation | 14.1% | Help with Independent Living | 13.4% |
| 6 | Social Support | 13.7% | Smoking Support | 14.1% | Befriending & Social Isolation | 13.0% |
| 7 | Community Activities - General | 13.3% | Help with Independent Living | 13.3% | Social Support | 12.7% |
| 8 | Financial and Benefits Support | 13.0% | Alcohol & Substance Misuse Support | 12.9% | Alcohol & Substance Misuse Support | 12.4% |
| 9 | Help with Independent Living | 12.3% | Social Support | 12.4% | Carer Support | 12.2% |
| 10 | Alcohol & Substance Misuse Support | 12.0% | Community Activities - General | 12.1% | Community Activities - General | 12.2% |
| 11 | Learning and Development | 12.0% | Carer Support | 11.9% | Financial and Benefits Support | 12.1% |
| 12 | Carer Support | 11.7% | Financial and Benefits Support | 11.7% | Smoking Support | 11.8% |
| 13 | Housing Support | 10.3% | Housing Support | 11.4% | Housing Support | 10.7% |
| 14 | Smoking Support | 10.3% | Learning and Development | 10.4% | Learning and Development | 10.7% |
| 15 | COVID-19 | 2.3% | Weight Management | 2.9% | Weight Management | 1.9% |
